# Annotations

**Published:** 1895-09-14

**Authors:** 


### Annotations.

### A Hospital for Coatbridge.

Some interest attaches to the fact that the late Mr. John Alexander has left £30,000 to build and endow a hospital for the benefit of the town of Coatbridge and its neighbourhood, in that it will inform people that there exists in the middle ward of Lanarkshire a town which has grown with something like American suddenness. Thirty years ago Coatbridge was only a village; to-day it ranks seventh among the towns of Scotland in point of population, having outgrown the ancient city of Perth in the last decade. Yet its development is owing only to the sudden expansion of an old industry. Long centuries ago the pious occupants of "the Monklands," in which the town of Coatbridge has grown, dug coal from the earth around them; but the resources of modern civilisation have developed a small industry into a great one, and made the ploughmen of a generation ago the millionaires of to-day. Steel works have been added to coal mines, till the town of Coatbridge stands out like the guiding beacon of the Israelites, "a pillar of smoke by day and a pillar of fire by night." Yet for a large population, the majority of them exposed, by the very nature of their occupation, to exceptional risks and dangers, there has been hitherto no hospital accommodation, and broken limbs and burnt bodies had either to be treated at home or jolted in agony to Glasgow. Mr. Alexander had grown with the town, and its interests were his. He was a self-made man, who owed the wealth he possessed to the development of the coal and iron industry, and various gifts to the town he lived in showed how he identified himself with its growth and welfare. He was, perhaps, a hard man in appearance, not one to express an easy sympathy with every grief, nor be imposed upon by specious troubles. Keen, shrewd, and suspicious, as an employer of labour is almost compelled to be, he yet cared for those who worked under his direction, and this last donation will perpetuate his name among those who were most closely connected with him during his life—those to whom, in a sense, he owed his wealth, but who again earned their living through him, and who now will look back on him with affection and gratitude.

### The Powers of the Ship's Surgeon.

We have received a pamphlet entitled "Ship Owners and Ship Surgeons." Dr. Leet's contention is that the medical officers of the British Merchant Navy are absolutely impotent as sanitary officers afloat, that neither captain nor owner listens to their complaints, and that, while they may doctor passengers and crew when these are ill, they are not permitted to offer any opinion as to the conditions which largely go to determine sickness and health, such as ventilation, food, sanitary appliances, and the like. Dr. Leet, for speaking his mind on these matters, has, he says, had his services dispensed with by "three of the foremost shipping companies." Moreover, now that he has settled down to practice on shore, the employés of these companies have been warned that it will be injudicious for them to employ him. This is a serious charge, and we regret that Dr. Leet has not thought it wise to substantiate it by naming the companies. For ourselves, we think the accusations may be just. It is likely enough that even in the big passenger lines, where everything is done for the comfort of passengers, the sailors' accommodation may be cramped in space and defective in ventilation. If that be so it is only too probable that the "impecunious and unscrupulous private owner" may do still worse by his mariners. But if Dr. Leet knows sure instances of this, why not name them? From his own statement he has nothing more to lose by the malignity of ship owners (for surely he does not fear they will attempt his life), and in any real grievance of our sailors he may be sure that the British public will support him. We think, too, that his suggestion that the ship's surgeon should have powers similar to those of a medical officer of health on land is reasonable. If the doctor, the expert, has not the supreme say on matters of health, who should have? But we have no sympathy with these anonymous accusations. Let him name the criminals, and he may be sure of both support and sympathy.

### A School of Economics.

There is no science in which the general public is so much interested as economics, and yet comparatively few know that it is a science at all. If one man succeeds in business and another fails, it is set down, at least by the man who fails, to some mysterious luck, and not to his superior knowledge. If one is not prepared to say that the struggling millions can be transformed into millionaires by a sudden change in the forms of government, he is liable to be called a brute—to use the mildest term—by people who know nothing of what they are suggesting. Popular revolutions and political follies are the result of the general ignorance of the conditions which regulate the acquisition and distribution of wealth, and anything that tends to clear away that ignorance is greatly to be desired. Therefore we welcome the proposed institution of a School of Economics in London, where lectures will be given, and classes will be held, on the history of commerce, on banking, on the effect of alien immigration, on trade routes and railways, on the theory of taxation, on everything that concerns money and its equivalents. There is a growing conviction that there must be a "reason why" for wealth and poverty, as well as other things, but false and specious reasons are so often accepted as true, that it is well to diffuse a general knowledge of the principles which underlie the great money question. The course sketched out by the London School of Economics will not show every man how to get rich, for the great money-maker is, like the poet, born, not made. But some sound knowledge of finance may save many men from losing money, which, perhaps, contributes more largely to the general weal. Therefore we commend, as an indication of public wisdom, the establishment of this school, which is to be held at 9, John Street, Adelphi, W.C., under the direction of Mr. W. A. S. Hewins, M.A., Pembroke College, Oxford, and Donkin Lecturer at Manchester College, Oxford.

